# Bioengineering of CuO porous (nano)particles: role of surface amination in biological, antibacterial, and photocatalytic activity

**DOI:** 10.1038/s41598-022-19553-2

**Published:** 2022-09-12

**Authors:** Mojtaba Bagherzadeh, Moein Safarkhani, Amir Mohammad Ghadiri, Mahsa Kiani, Yousef Fatahi, Fahimeh Taghavimandi, Hossein Daneshgar, Nikzad Abbariki, Pooyan Makvandi, Rajender S. Varma, Navid Rabiee

**Affiliations:** 1 Department of Chemistry, Sharif University of Technology, Tehran, Iran; 2 School of Engineering, Macquarie University, Sydney, NSW 2109 Australia; 3 Nanotechnology Research Centre, Faculty of Pharmacy, Tehran University of Medical Sciences, Tehran, 14155-6451 Iran; 4 Department of Pharmaceutical Nanotechnology, Faculty of Pharmacy, Tehran University of Medical Sciences, Tehran, 14155-6451 Iran; 5 Universal Scientific Education and Research Network (USERN), Tehran, 15875-4413 Iran; 6 Istituto Italiano di Tecnologia, Centre for Materials Interfaces, Pontedera, 56025 Pisa, Italy; 7 Regional Centre of Advanced Technologies and Materials, Czech Advanced Technology and Research Institute, Palacky University, Olomouc, Slechtitel, ů 11, 783 71 Olomouc, Czech Republic; 8 Department of Materials Science and Engineering, Pohang University of Science and Technology (POSTECH), 77 Cheongam-ro, Nam-gu, Pohang, Gyeongbuk 37673 South Korea

**Keywords:** Biocatalysis, Bioinorganic chemistry

## Abstract

Nanotechnology is one of the most impressive sciences in the twenty-first century. Not surprisingly, nanoparticles/nanomaterials have been widely deployed given their multifunctional attributes and ease of preparation via environmentally friendly, cost-effective, and simple methods. Although there are assorted optimized preparative methods for synthesizing the nanoparticles, the main challenge is to find a comprehensive method that has multifaceted properties. The goal of this study has been to synthesize aminated (nano)particles via the *Rosmarinus officinalis* leaf extract-mediated copper oxide; this modification leads to the preparation of (nano)particles with promising biological and photocatalytic applications. The synthesized NPs have been fully characterized, and biological activity was evaluated in antibacterial assessment against *Bacillus cereus* as a model Gram-positive and *Pseudomonas aeruginosa* as a model Gram-negative bacterium. The bio-synthesized copper oxide (nano)particles were screened by MTT assay by applying the HEK-293 cell line. The aminated (nano)particles have shown lower cytotoxicity (~ 21%), higher (~ 50%) antibacterial activity, and a considerable increase in zeta potential value (~ + 13.4 mV). The prepared (nano)particles also revealed considerable photocatalytic activity compared to other studies wherein the dye degradation process attained 97.4% promising efficiency in only 80 min and just 7% degradation after 80 min under dark conditions. The biosynthesized copper oxide (CuO) (nano)particle’s biomedical investigation underscores an eco-friendly synthesis of (nano)particles, their noticeable stability in the green reaction media, and impressive biological activity.

## Introduction

Nanoscale metal-based materials has found its equitable place in daily human life, and its impact is quite evident in science and industry^1–3^. Copper oxide nanoparticle is one of the essential materials with various applications in chemical reactions and biochemical processes. Copper oxide and copper-based nanoparticles have shown biological activity (e.g., antibacterial, antioxidant, and anticancer) and accordingly is used in biomedical sector along with environmental applications^4–7^. These compounds are utilized in the tissue engineering^8^, gene therapy^9^, disease diagnostics^10^, and target cancer therapy^11–13^.

In nanomedicine, there are numerous advantages in employing plants as natural and renewable resources for the green synthesis of metal nanoparticles. Such an ideal approach for the nanofabrication of CuO brings value and may pave the way for biomedical applications. In such biological processes, the toxicity of utilized reagents for the nanoparticle's synthesis is relatively low, and natural elements are deployed for reducing, capping, and stabilizing the metal’s nanoparticles. Natural materials such as plants and agricultural residues and waste encompassing compounds like quinines, alkaloids, terpenoids, flavonoids, fatty acids, enzymes, amino acids, phenols, and tannins have distinctive roles in the synthesis of nanoparticles. The biological method is environmentally friendly and leads to highly efficient and cost-effective synthesis and has been extensively used in assorted applications such as drug delivery, gene therapy, nanobiotechnology, medicine, biomedical engineering, and pharmacology^14–19^.

Although chemical and physical synthesis methods have been vastly utilized for the preparation of nanoparticles, being non-eco-friendly, and involving hazardous material usage diminishes their acceptance. In this regard, the bioengineered synthesis methods could replace these methods as they exploit plant phytochemicals or microbial enzymes. Among biologically synthesized nanoparticles (graphene oxide, iron oxide, zinc oxide, platin, selenium, gold, silver), CuO nanoparticles have garnered attention because they comprise a co-factor of the plethora of human neuropeptide enzymes, and are involved in immune cell function, anti-oxidant defense, and cell signaling regulation^20^.

The bioengineering synthesis process can be divided into three main categories (i) using plant and plant extract (phyto route), (ii) utilizing microorganisms like actinomycetes, bacteria, yeasts, and fungi (microbial pathway), and (iii) usage of templates such as diatoms, viruses, and membrane (bio-template route). The pathway utilizing the abundant plant extractives is preferred to other means because of greener attributes and being well dispersed, endowed with a faster pace of synthesis and relatively lower cytotoxicity; the plant extracts can provide electrons that help reduce the copper salt and also serve as stabilizing agents^21^. Some of the major contributions to the assembly of CuO nanoparticles via a bioengineered pathway are summarized in Table 1.

**Table 1:** Bio-engineeringly synthesized CuO nanoparticles with various plants extract.

Metal salt	Plant	Size (nm)	Features	References
CuSO_4_	*Sida acuta* Burm	50	Crystalline	^22^
CuSO_4_	*Adiantum lunulatum* Burm	10	Quasi-spherical	^23^
CuSO_4_	*Bauhinia tomentosa*	30	Spherical	^24^
CuSO_4_	*Piper* *betle*	75	Spherical	^25^
CuSO_4_	*Enicostemma littorale* Blume	30	Spherical	^26^
CuSO_4_	*Phoenix dactylifera*	25	Spherical	^27^
CuSO_4_	*Aloe barbadensis* Mill	20	Spherical	^28^
CuSO_4_	*Zea mays*	50	Spherical	^29^
CuSO_4_	*Vitis vinifera*	35	Spherical	^30^
CuSO_4_	*Ziziphus mauritiana* Lam	35	Spherical	^24^
CuSO_4_	*Coffea arabica*	260	Crystalline	^31^
CuSO_4_	*Gymnema sylvestre*	170	Spherical	^24^
CuSO_4_	*Glycine max*	20	Spherical	^32^
CuSO_4_	*Zingiber officinale* Roscoe	30	Spherical	^33^
CuSO_4_	*Inula helenium*	35	Spherical	^34^
CuSO_4_	*Syzygium aromaticum*	30	Granular nature	^35^
CuSO_4_.5H_2_O	*Solanum lycopersicum*	30	Spherical	^36^
CuSO_4_.5H_2_O	*Citirus medica*	20	Crystalline	^37^
CuSO_4_.5H_2_O	*Bacopa monnieri*	35	Spherical	^38^
Cu (NO_3_)_3_.6H_2_O	*Populus ciliate*	55	Spherical	^39^
Cu (NO_3_)_2_.3H_2_O	*Drypetes sepiaria*	300	Spherical	^40^
Cu (NO_3_)_2_.3H_2_O	*Abutilon indicum*	20	Spherical	^41^
CuCl_2_.2H_2_O	*Saraca indica*	50	Spherical	^42^
CuCl_2_.2H_2_O	*Psidium guajava*	15	Spherical	^32^
CuCl_2_.2H_2_O	*Ginkgo biloba*	20	Spherical	^43^
Cu (CH_3_COO)_2_.H_2_O	*Leucaena leucocephala*	12	Spherical	^44^
Cu (CH_3_COO)_2_.H_2_O	*Arachis hypogaea*	40	Spherical	^45^
Cu (CH_3_COO)_2_.H_2_O	*Ferulago angulata*	45	Spherical	^46^
Cu (CH_3_COO)	*Eclipta prostrata*	40	Spherical	^47^
Cu (CH_3_COO).2H_2_O	*Aloe vera*	40	Spherical	^48^

In the past few decades, various bacterium and viral disease have been treated with metals/metal oxide nanoparticles wherein CuO nanoparticles have displayed considerable antibacterial activities; CuO nanoparticles being highly toxic to the plethora of plant or human bacterial pathogens^49^ in view of their high chemical and biological reactivity, biocompatibility, high surface area, and small size^50^. When CuO nanoparticles come in contact with bacterium cells with help of amines and carboxylic groups on cell membrane, an effortless entry inside the cell occurs with the development of several malfunction cytotoxicity^51^. CuO nanoparticles generate ROS (reactive oxygen species) that can disrupt the membrane and interfere with cell division, metabolism, and DNA replication besides the degradation of ribosomes and mitochondria promoted by CuO nanoparticles-mediated cytotoxicity. The large redox potential of Cu leads to the generation of the Cu ions and these highly toxic ions accumulate the hydroxyl and superoxide radicals (oxidative stress)^52^. Chtita et al.^29^ fabricated a CuO nanoparticle utilizing *Gloriosa superba* leaf extract that revealed good inhibitory against *Klebsiella aerogenes* (gram-negative bacteria) and *Staphylococcus aureus* (gram-positive bacteria)^53^. *Sida acuta* leaf extracts has been used by Sathiyavimal and colleagues for the fabrication of CuO nanoparticles as they applied these biosynthesized compounds in the cotton fabrics against gram-negative and gram-positive bacterium with promising results^22^. Green synthesized CuO nanoparticles from the precursor, Cu (CH_3_COO)_2_ by Nwaya et al. demonstrated promising growth inhibitory activity against various species of pathogenic bacteria such as *Pseudomonas aeruginosa* and *Bacillus licheniformis*^29^*.*

One of the new era’s catastrophic problems is water pollution which has been a major concern for society; it has garnered much attention for the purification of wastewater. Unique features of nanomaterials make them perfect candidates for the degradation of water pollutants like dyes as these photocatalysts are efficient, inexpensive, and offer sustainable approach in wastewater treatment. Photocatalysis is based on having a band gap between the valence band (VB) and the conduction band (CB). When the excited electrons travel from VB to CB, electron–hole (e^−^/H^+^) pairs are generated and transferred to the surface of the catalyst and react with other materials such as O_2_ and H_2_O; In VB and CB, e^−^ and H^+^ are able to generate hydroxyl radical (·OH) and O_2_^−^, respectively which are responsible for the degradation of pollutants^54–56^. For example, Iqbal and coworkers synthesized CuO nanoparticles using an aqueous extract of *Rhazya stricta* and investigated it in the degradation of methylene blue (MB) wherein CuO NPs caused 83% degradation of MB after 140 min of reaction^57^. Some of the unique studies are listed in Table 3.

In this study, we have focused on the synthesis and characterization of CuO (nano)particles utilizing the *Rosmarinus officinalis* leaf extract. Besides, their photocatalytic and biological activity including the cytotoxicity and antibacterial assessment against *Bacillus* as a Gram-positive and *Pseudomonas* as a Gram-negative bacterium, was evaluated.

## Materials and methods

### Reagents, chemicals, and plant source

All reagents and materials have been of analytical grade and purchased from Sigma-Aldrich. The *Rosmarinus officinalis* was obtained from Kurdistan province in Iran, and all of the National Laws and/or protocols have adhered appropriately. Amir Mohamad Ghadiri collected the plant samples, and obtained the local permissions. A voucher specimen has been deposited in the herbarium of Prof. M. Bagherzadeh’s Lab at the faculty of chemistry of Sharif University of Technology, Tehran, Iran (Deposition N.O: A.M.G.1581ROSMARI). Although, the plant *Rosmarinus officinalis* has been well studied in the other studies by the GC–MS technique, identifying this plants ingredients need more studies^58,59^. The authors confirm that all methods were performed in accordance with the relevant guidelines and regulations.

### The plant extract preparation

The *Rosmarinus* *officinalis* was washed with distilled water and then kept at room temperature to dry^20^. The powdery form of the dried plant was prepared by grinding, and the fine pulverized powder (10 g) was dispersed in deionized water (100 mL) and placed for 15 min on a heater stirrer at the boiling point of the solvent and then, set at room temperature for cooling. The Whatman filter paper (grade one) used for filtration of the final solution and the prepared extract have been stored for further experiments at a 4 °C^46,60,61^.

### Synthesis of CuO (nano)particles

**Figure 1:**
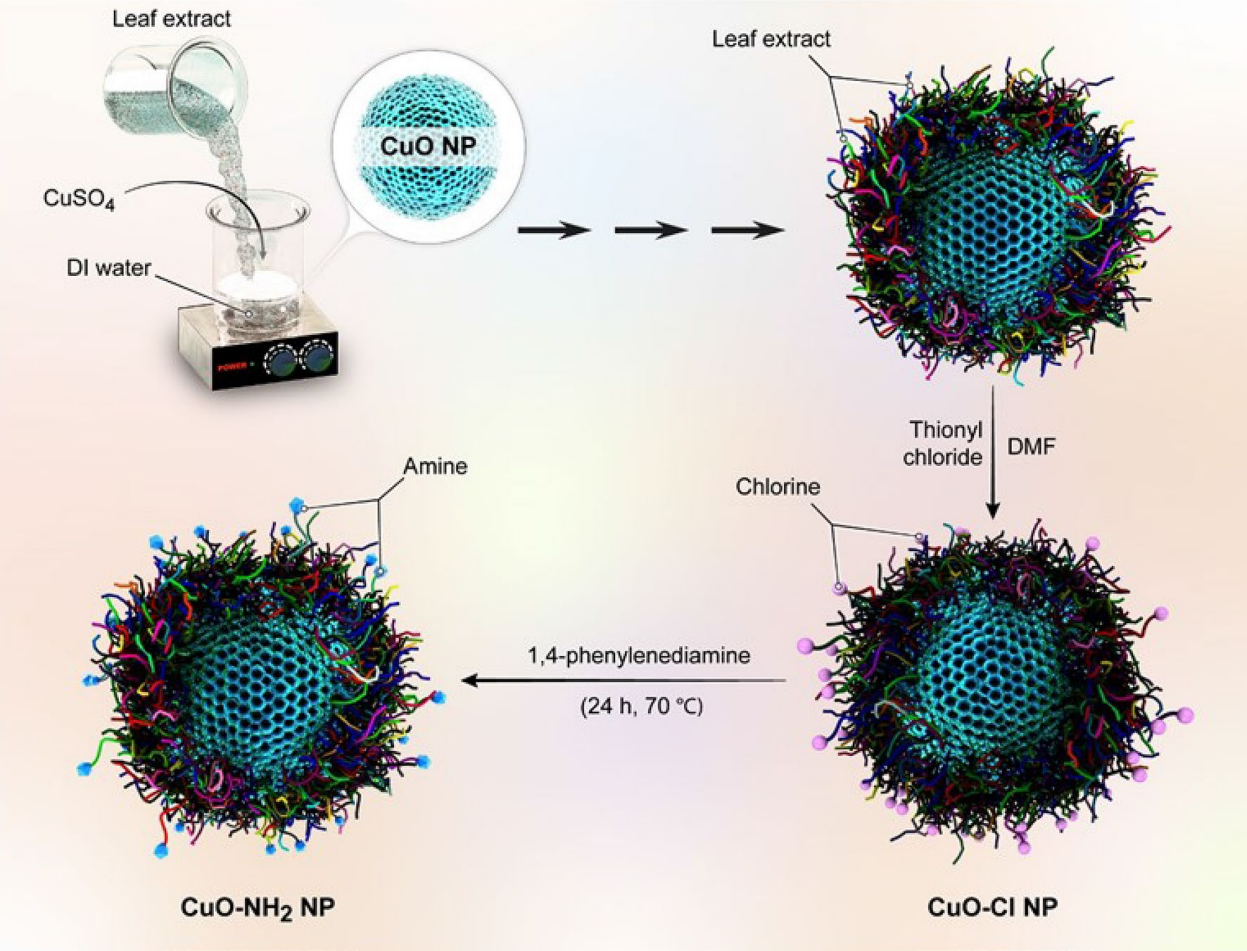
Schematic illustration for the synthesis of CuO.

For the synthesis of copper oxide (nano)particles from the leaves of *Rosmarinus officinalis*, 40 mL of extract of *Rosmarinus officinalis* was transferred to the cupric sulfate solution (160 mL, 1 mM), and after a while, the color changed from mild blue to dark one; mixture being agitated at 25 °C for 24 h. The leaf extract played two crucial roles in this study, as a stabilizing agent and a reducing agent wherein the capping of (nano)particles with ketone and aldehyde groups can control the growth, aggregation, and also reduction process; plant extract has a huge impact on the shape, size, and morphology of the ensued (nano)particles. Based on reported data, higher the concentration of the plant extract, the smaller is the size of ensued (nano)particle, which is a consequence of the presence of additional phytoconstituents. The product was characterized by different techniques such as FT-IR, UV–Vis, and PXRD (Fig. 1).

For separation of unreacted materials and byproducts from the reaction mixture and the CuO (nano)particles, the solution was centrifuged for 25 min at 10,000 rpm and then washed with deionized water and ethanol (three times). The copper oxide-dried powder was obtained by freeze-drying. The technique of ultra-centrifugation was utilized to separate (nano)particles based on their size^62–64^.

### Chlorination of copper oxide (nano)particles

Chlorine is one of the most promising ligands in coordination chemistry, which facilitates further functionalization. Accordingly, 15 mL of thionyl chloride was added to the filtered-off product (CuO (nano)particles); additionally, to increase the rate of reaction, 2 mL of DMF was added (18 h, yellow solid, argon atmosphere). Then to obtain a dried powder, the solution was kept in the oven for 24 h.

### Amination of functionalized copper oxide (nano)particles

Based on the recent studies, the amination process would invariably lead to lower toxicity and increase the zeta potentials. To accomplish this, 50 mg of the chlorinated product of copper oxide (nano)particles were added to 10 mL DMF in 70 °C on stirrer, then 50 mg of 1,4-phenylenediamine was added to the mixture. The final solution was stirred for 24 h at 70 °C.

### Characterization of CuO (nano)particles

The JASCO FT-IR-460 spectrometer has been utilized for Fourier transformed infrared spectroscopy (FT-IR) in the field of 400–4000 cm^−1^. An automated Philips X'Pert X-ray diffractometer obtained powder X-ray diffraction (PXRD) spectra with Cu K radiation (40 kV and 30 mA) for 2θ values over the range of 10–80. The synthesized (nano)particle morphology and elemental analysis (FESEM, EDS, and map) have been observed under an acceleration voltage of 30–250 by a field emission scanning electron microscope (TESCAN MIRA-3). To record the (UV–Vis) spectra at the range of 200–800 nm, the Perkin Elmer Lambda 25 has been utilized. The nanoparticle size was screened by (DLS) analysis and (Horiba SZ100). The fluorescence spectrometer (PerkinElmer, USA) was utilized for recording (PL analysis).

### Antibacterial activity

Antibacterial activities of the compounds against *Bacillus cereus* and *Pseudomonas aeruginosa* were assessed by the renowned disk diffusion method utilizing Müller Hinton agar and Sabouraud Dextrose Agar (SDA). The inhibition zone on the incubation completion has been recorded, and the average diameter for every compound was recorded at 400 μg mL^−1^, and the dimethyl sulfoxide (DMSO)-based Stock solutions of compounds were prepared. Standard antibiotics like penicillin, ampicillin, and gentamicin with similar concentrations have been utilized to compare with compound inhibition zone. To minimize the error, each test was carried out several times (at least three times). As the effect of the DMSO at the biological screening should be clarified, blank studies have been accomplished, and no activity was observed in pure DMSO against any bacterial strains^65,66^.

### Photocatalytic activity

The photocatalytic activity of CuO–NH_2_ (nano)particles was evaluated in an aqueous solution by calculation of methylene blue degradation. For this purpose, the utilized light source was a 250-W mercury lamp to prepare visible light with a wavelength of more than 420 nm. An optical glass (400–800 nm cutoff filter) was used to determine photocatalytic activity. The photocatalytic tests were carried out with a 100 mL photoreactor at STP^67^. Every analysis was performed using 0.3 g/L of dispersed CuO–NH_2_ as the photocatalyst in the 10 mg/L methylene blue aqueous solution. The mixture was stirred and exposed under irradiation simultaneously. To calculate the methylene blue concentration, at the regular steps (exact time interval), 4 mL of solution was separated and screened at 600 and 670 nm (absorbance wavelength)^68,69^.

### MTT assay

The cytotoxicity of synthesized (nano)materials has been investigated by applying the HEK-293 cell line. In brief, 100 µL of F12/DMEM was supplemented with ten percent FBS and incubated in a 96-well plate seeded with 10^5^ cell density per well. The fresh media of several diluted prepared (nano)particles replaced the culture media then newly prepared cells were incubated for 5 h. As mentioned above, the media is replaced with fresh media (for 24 h) at the next stage. After four h incubation at STP, the prepared media aspirated and, in this stage, generated MTT formazan has been dissolved in Dimethyl sulfoxide. Every well’s absorbance has been recorded at 570 nm using a microplate reader^70–73^.

### Leaves collection permission

The *Rosmarinus officinalis* have been obtained from Kurdistan province in Iran, and all of the National Laws and/or protocols have adhered appropriately. Amir Mohamad Ghadiri collected the plant samples, and obtained local permissions.

## Results and discussion

### Synthesis and characterization

**Figure 2:**
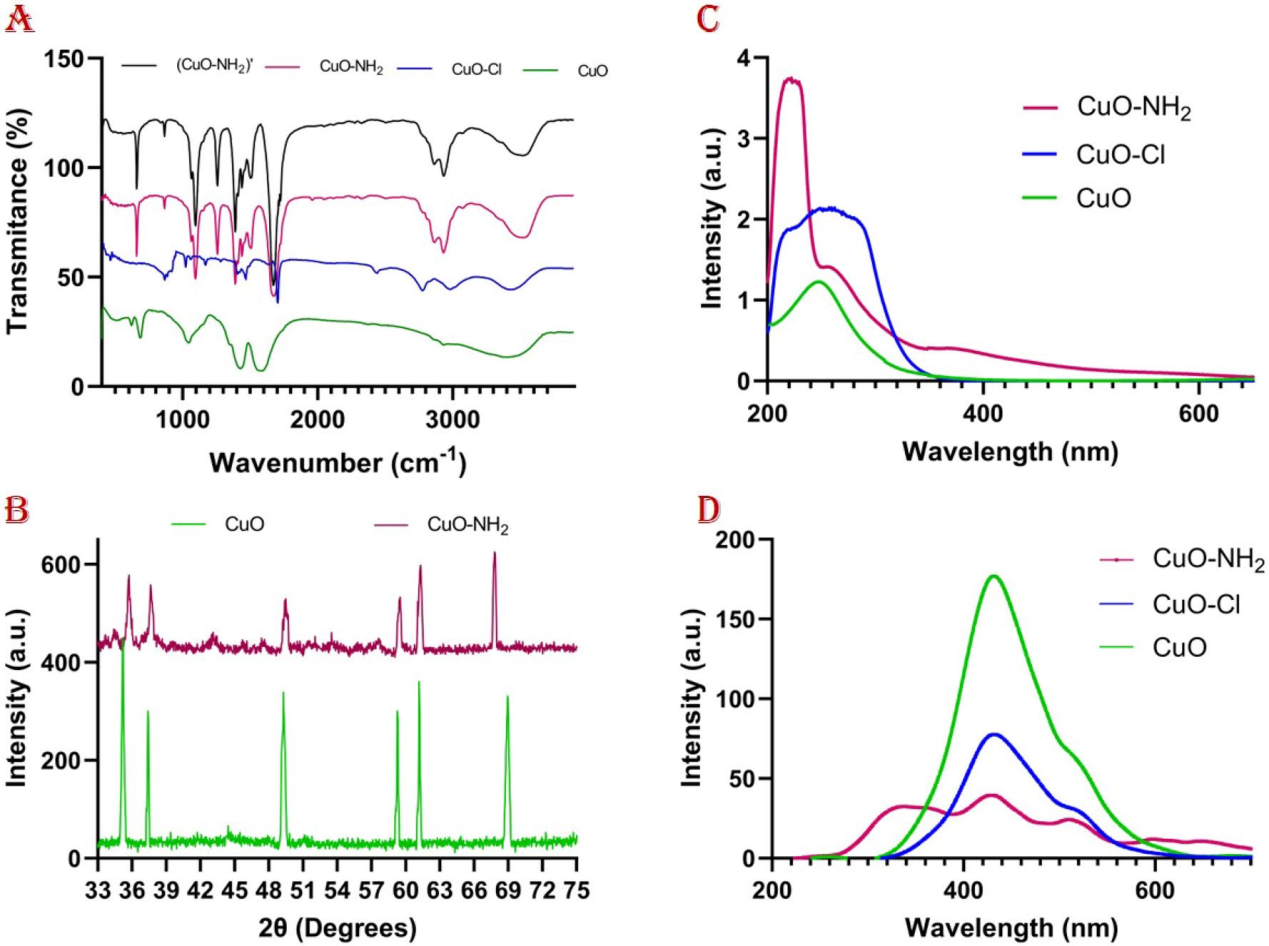
(**A**) The FT-IR spectra, (**B**) the PXRD, (**C**) the UV–Vis spectra, and (**D**) the photoluminescence spectra of prepared (nano)particles.

Considering the FT-IR spectrum (Fig. 2A), the broad bands are revealed at ~ 3380 cm^−1^ representing the hydroxyl group stretching frequency (hydroxyl groups on the surface of the CuO (nano)particles). The Cu–O bonds stretching vibration indicator has been revealed^74,75^ on the 686 cm^−1^. The peak at 2925 cm^−1^ proved the presence of carboxylic acid O–H stretching, while another one at 1566 cm^−1^ is for aliphatic nitro compounds. In CuO–Cl spectra, a band at about 870 cm^−1^ belongs to C–Cl. Also, the peak at 2421 cm^−1^ (thiol S–H stretch) is not observed in CuO–NH_2_ spectra, which showed the replacement of amide and thiol on the surface of copper oxide (nano)particles. Additionally, in CuO–NH_2_ spectra, the bands observed at ~ 2900 cm^−1^ is accredited to N–H bonding contributed by 1,4-phenylenediamine on CuO (nano)particles. Their stability after photocatalysis process is affirmed by the subsequent FT-IR analysis of CuO–NH_2_ (nano)particles as depicted in the Fig. 2A which is reliable evidence of (nano)particles stability (Table 2)^62,76–80^.

**Table 2:** The survey on the recent advancements on the biomedical potentials of the CuO nanoparticles.

Plant	Functional group (cm^−1^)	Shape	Size (nm)	UV–Vis (nm)	Antibacterial activity	Diffraction peaks (2θ°) or Bragg’s reflection	Photocatalytic activity	ref
Eupatorium odoratum	(O–H, 3976) (C–H, 2936) (C=O, 1618)	Spherical	12–30	211, 305	*S. aureus, B. cereus, E. coli*	–	−	^92^
Kalopanax pictus	(N–H, 3467) (C=C, 1584) (C–N, 1360)	Spherical	26–67	368	*–*	–	+	^13^
Eichhornia crassipes	(O–H, 3314) (N–H, 1624) (C–O–C, 1217)	Spherical	15–30	310	*Aspergillus flavus, niger, fumigatus*	–	−	^74^
Oak	(3415, O–H) (1654, C=O)	Quasi-cubic	34	590	*–*	(110), (− 111), (111), (− 202), (020), (202), (− 113), (− 311), (220), (004)	+	^93^
Terminalia catappa L	(3209, O–H) (2920, C–H) (1557, C=O)	Spherical	29–103	215, 260 372	*–*	(110), (112), (202), (220), (004)	+	^94^
Euphorbia pulcherrima	(3384, O–H) (1595, C=O)	Cubic	19	240	*–*	(110), (002), (111), (202), (020), (202), (11–3), (31–1), (113), (004)	−	^95^
Rosa canina	(3200–3550, O–H) (1670, C=O) (1405, C=C)	Spherical	15–25	262	*–*	(110), (111), (200), (202), (020), (202), (113), (311), (220), (400)	−	^44^
Calotropis procera	(3414, O–H) (2923, C–H) (1598, C=C)	Cylindrical	46	291, 355	*–*	(100), (002), (200), (202), (020), (202), (113), (311), (220), (222)	−	^89^
Sambucus nigra	(3300–3500, O–H) (2299, C–H) (1621, C=C)	–	–	270	*–*	36, 39, 49, 54, 59, 62, 67, 69, 73, 75	−	^96^
Punica granatum	(3379, O–H) (1577, C=O)	Spherical	10–100	282	*–*	35, 38, 48, 52, 56, 61, 65, 74	+	^97^
Aloe barbadensis	(3405, O–H) (1538, C=C) (944, C–C)	Spherical	15–30	265, 285	*–*	(110), (111), (200), (202), (020), (202), (113), (311), (220), (400)	−	^93^
Sida Rhombifolia	(3439, O–H) (1658, C=C)	Spherical	10	260, 321	*E. coli, Klebsiella pneumonia* and *Pseudomonas*	33, 35, 38, 49, 53, 57, 63, 66, 67	+	^44^

“− “ and “+ ” represent the absence and presence of the activity.

The structure of the synthesized CuO has been investigated by means of PXRD (Fig. 2B). The six prominent characteristic diffraction peaks for CuO are around 2θ = 34.5°, 37.6°, 48.7°, 58.8°, 60.8°, and 69.8°, which corresponds to the (002), (111), (202), (202), (113), and (220) crystallographic planes (The prepared copper oxide nanoparticle matched with the previously recorded XRD pattern of CuO registered as (JCPDS card No. 04-0784), the fcc (face-centered cubic)). In CuO–NH_2_ (nano)particles’ PXRD spectra, there are other peaks around 2θ = 35°, 38°, 49°, 59°, 61°, and 69°. In the PXRD spectra, the impurities impact contributes to broadening the peaks. These diffraction peaks are in reliable agreement with other studies (Table 2)^81–84^.

PL spectroscopy has been widely applied to evaluate the rate of recombination of photogenerated electron–hole pairs on irradiated semiconductor nanoparticles^85^. One of the significant points is that the PL intensity has a direct relation with the electron–hole pair recombination rate. In general, it is well known that as the PL intensity becomes weaker, the recombination rate in semiconductors decreases; as a result, the lifetime of photogenerated charge carriers increases significantly, which causes the better photocatalytic activity of the desired photocatalyst. Figure 2D exhibits the PL spectra of CuO, CuO–Cl, and CuO–NH_2_ nanoparticles at the maximum excitation wavelengths of these nanoparticles (Fig. 2C). As can be clearly seen in the figure, the PL intensity spectrum of CuO–NH_2_ NPs is relatively lower than that of CuO–Cl and CuO nanoparticles. Also, the PL intensity spectrum of CuO–Cl nanoparticles is relatively lower than that of pure CuO nanoparticles. These results clearly illustrate that the electron–hole pair recombination can be significantly reduced by modifying the surface of copper oxide nanoparticles with chlorine and amine. The inhibition of electron–hole pair recombination by the surface amine group leads to a more efficient separation of photo-generated charge carriers, which eventually increases the photocatalytic activity of semiconducting NPs. Compared with pure copper oxide NPs and chlorinated copper oxide NPs, aminated copper oxide NPs exhibited a remarkable quenching in the PL intensity emission signal, which indicates that the amination of copper oxide NPs can lead to better separation efficiency of photo-generated charge carriers and consequently enhance the efficiency of photocatalytic activity of semiconducting nanoparticles.

**Figure 3:**
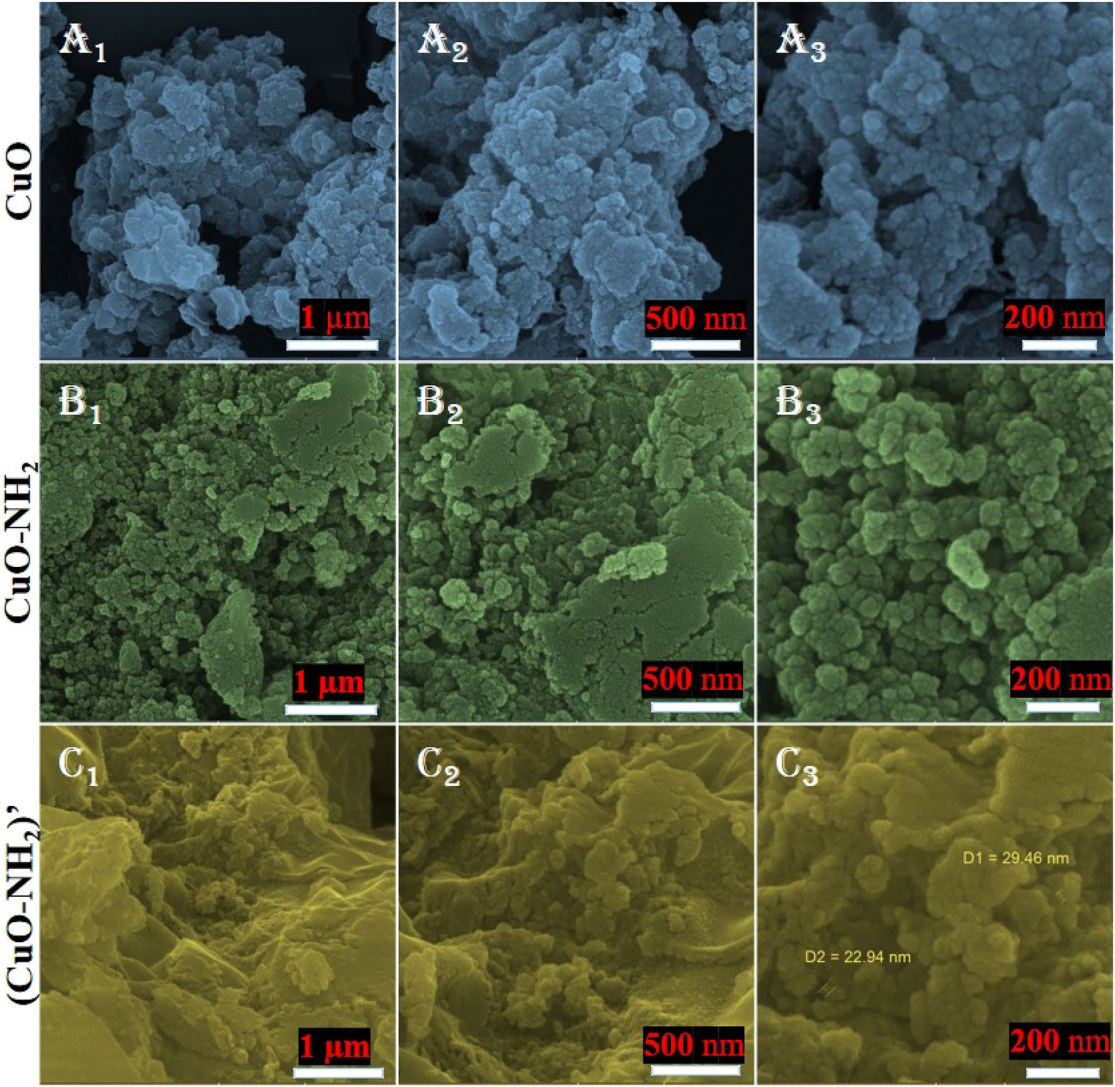
FESEM images of the synthesized CuO: (**A**_**1**_–**A**_**3**_), CuO–NH_2_: (**B**_**1**_–**B**_**3**_), and CuO–NH_2_ after photocatalysis process: (**C**_**1**_–**C**_**3**_).

The FESEM analysis has been utilized to evaluate the synthesized (nano)particle’s morphology. The FESEM images of the synthesized CuO (nano)particles mediated by *Rosmarinus officinalis* leaf are depicted in Fig. 3A1–A3, the aminated CuO (nano)particles being shown in Fig. 3B1–B3. The homogenous size range and monodispersed distribution are the features of these biosynthesized copper oxide (nano)particles. Stabilizing agents as well as reducing agents can change the morphology and the shape of (nano)particles both of them being performed by the plant extract in this case. The ensued data are in agreement with recent descriptions of copper oxide nanoparticles^78–80,86–88^. The Fig. 3C1–C3 is related to the CuO–NH_2_ (nano)particles after photocatalysis activity which illustrates that the surface morphology of prepared (nano)particles did not change considerably relative to the pristine CuO–NH_2_ surface morphology, which presents compelling evidence of (nano)particle’s stability. The DLS results demonstrate that the amination process can lead to increase of the particle size, and it could be due to increasing the hydrogen bands (Figs. S5–S7).

**Figure 4:**
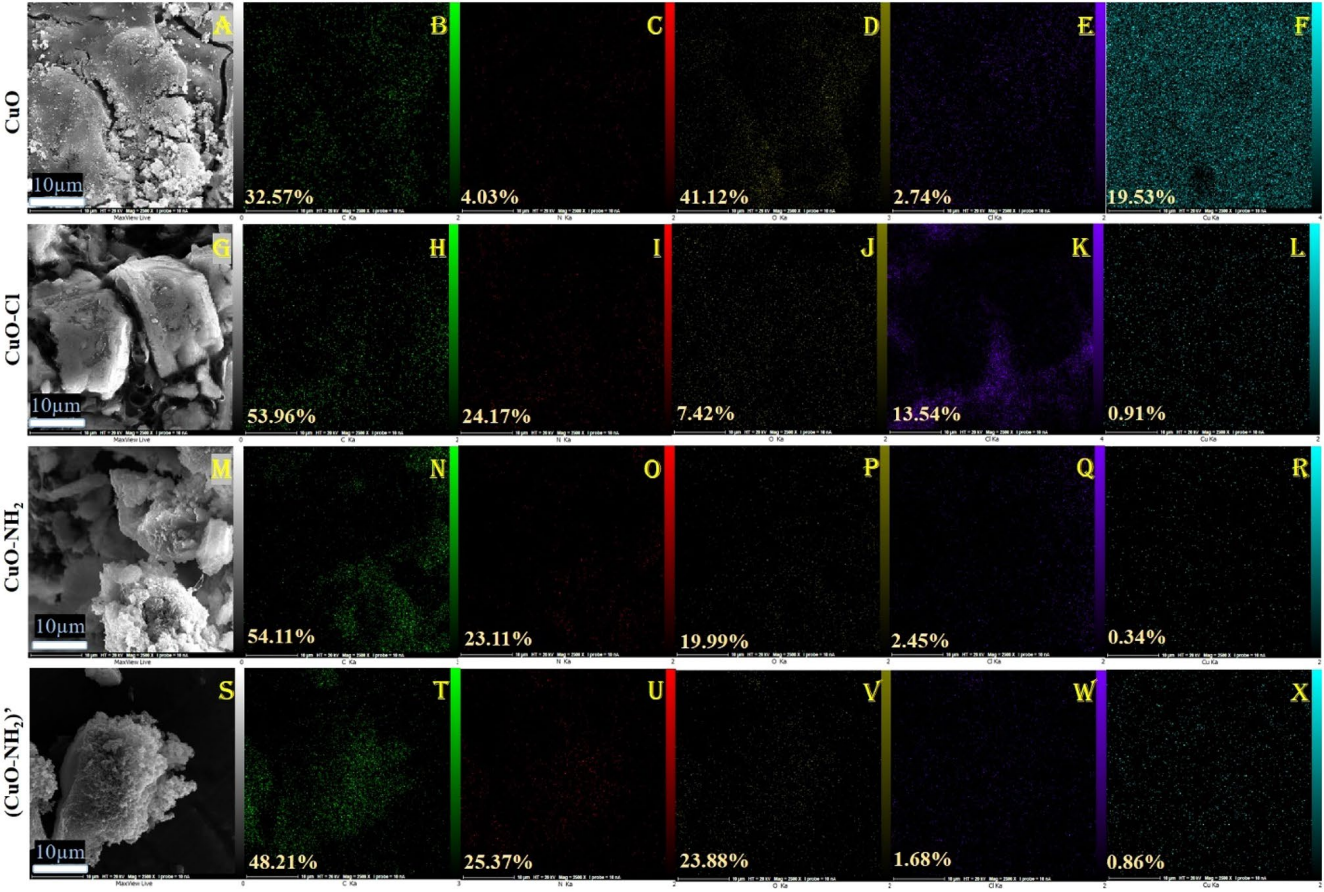
The EDS and mapping analysis of prepared nanoparticles, (**A**–**F**) CuO, (**G**–**L**) CuO–Cl, (**M**–**R**) CuO–NH_2_, and (**S**–**X**) CuO–NH_2_ after photocatalysis activity (CuO–NH_2_)’. Green: carbon, Red: nitrogen, Gold: oxygen, Violet: chlorine, and Blue: copper.

The elemental analysis (EDS) and elemental mapping are presented in Fig. 4A–X which revealed that all elements are well distributed on the (nano)particles’ surface, their percentage changing in considerable concordance with the synthesis steps. The zeta potential of prepared CuO and CuO–NH_2_ (nano)particles were − 8.4 and + 5 mV that; this considerable difference, was in good accordance with EDS results (elements’ percentage change) (Figs. S3 and S4).

### Antibacterial activity

**Figure 5:**
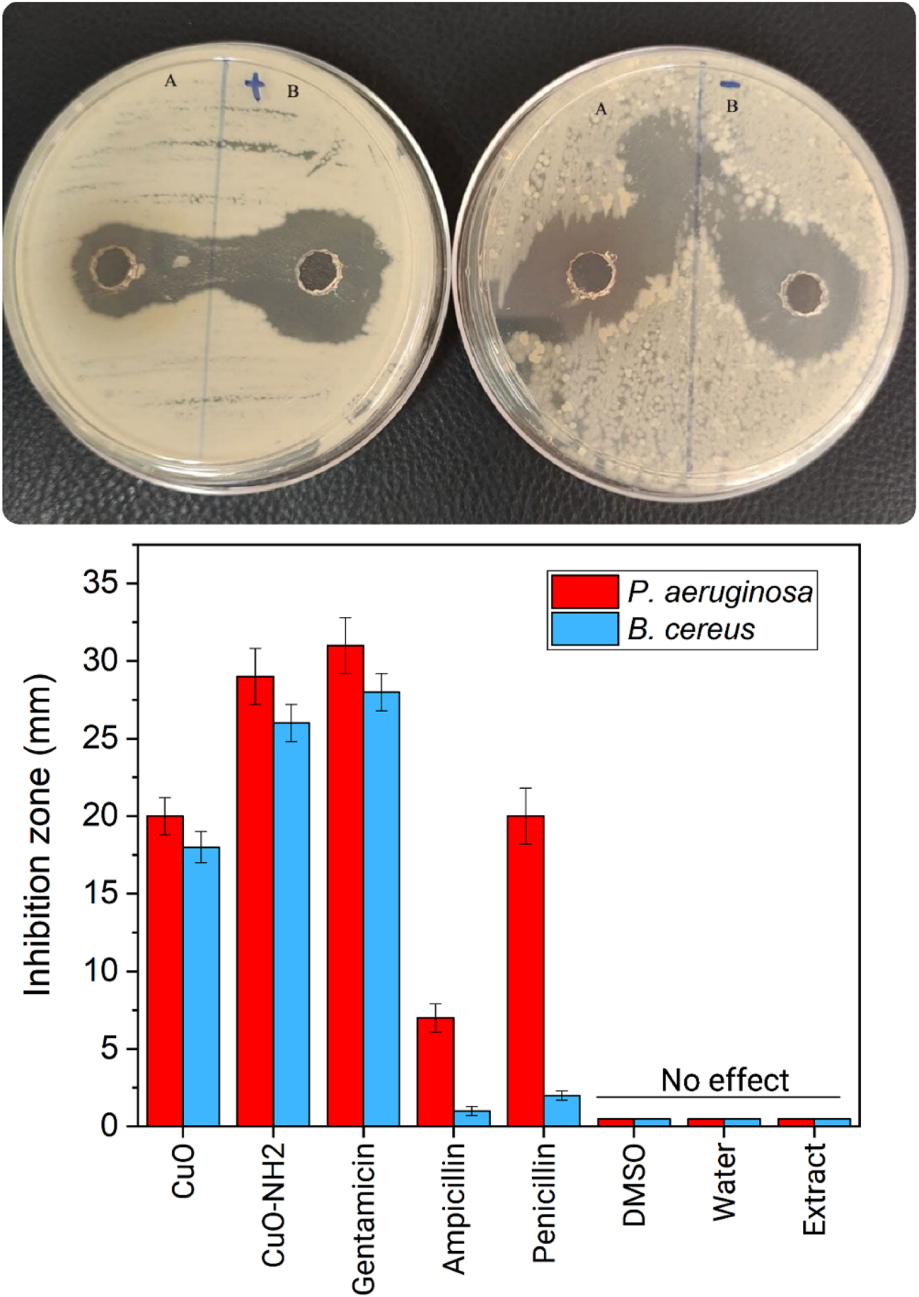
Antibacterial activity of the synthesized (**A**) CuO, (**B**) CuO–NH_2_ (nano)particles.

There are some probable mechanisms for antibacterial activity of metal nanoparticles such as disruption of cell membrane, DNA and protein damage, cell substances oxidation, attachment to ribosome, generation of ROS (reactive oxygen species), prevention of biofilm production, proton efflux damage, penetrating and then connection of metal ion to the cell’s sulfur and phosphorus which leads to apoptosis and connection of metal ion to the thiol group of the cell-surface protein. However, the precise mechanism is unknown, the further biological studies are necessary to broaden the available data. The prepared compounds have been tested for antibacterial activity against *Pseudomonas aeruginosa* gram negative species *and Bacillus cereus* as Gram positive. The amination of the CuO (nano)particles leads to ~ 50% increasing in antibacterial activity for example, the aminated CuO (nano)particles inhibition zone on *Pseudomonas aeruginosa* is about 29 mm while, the inhibition zone of CuO (nano)particles is 20 mm. The solvent and the extract of plant do not show momentous activity, it is proven that these compounds show antibacterial activity in comparison with that detected for standard antibiotic gentamicin, penicillin, and ampicillin. The results are compared with other researches and the collected data is quite considerable (Fig. 5). The ROS generated by CuO NPs can interact with bacteria’s cell membrane for penetrating to the cell and this connection can make some malfunction that inhibit the bacterial growth and leads to cell death. The smaller the size of (nano)particles, easier is the entry without any interference. The abundant functional groups such as amine and carboxyl on the surface of the cell can attract the Cu cations towards the cell. The CuO amination has shown enhancement in antibacterial activity. The metal–ligand linkage inertness presumably enlarges its protection against enzymatic degradation, cell permeability, and lipophilicity^89–91^.

### Photocatalytic activity

**Figure 6:**
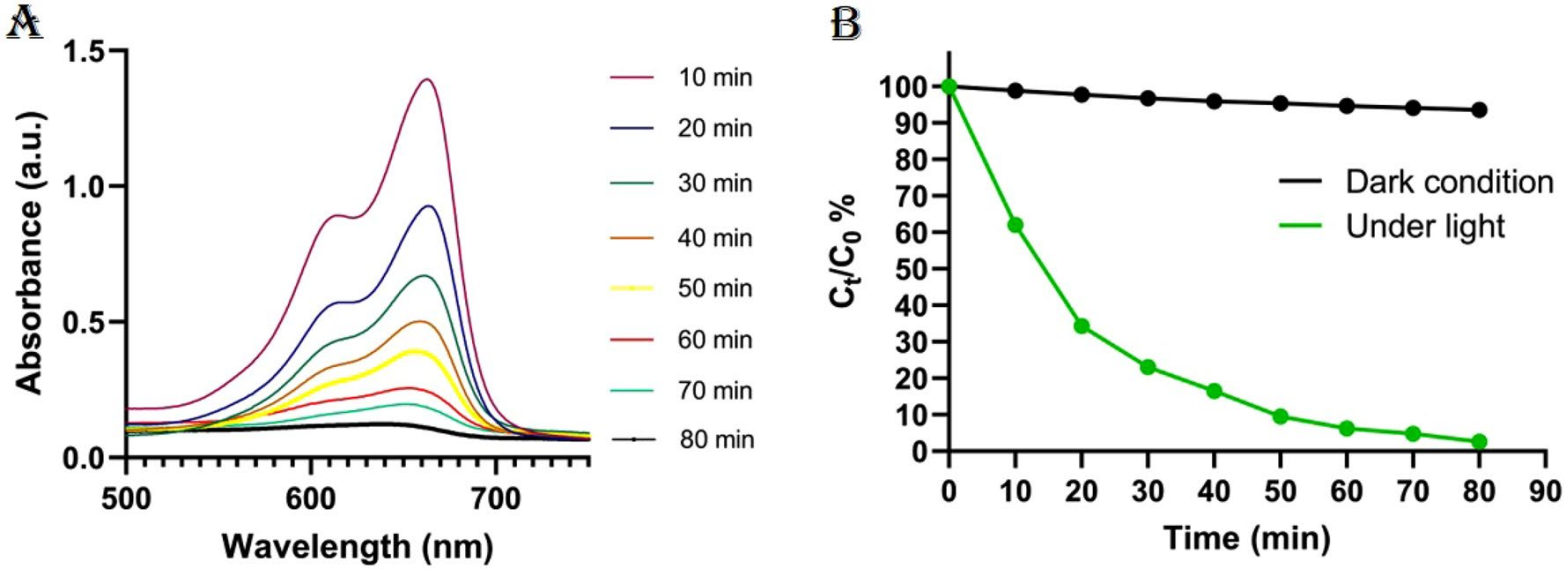
The CuO–NH_2_ (nano)particles (**A**) photocatalytic activity on the methylene blue and (**B**) the efficiency of photodegradation under light or dark condition.

The photocatalysis has been proposed for abatement the environmental pollutants and nowadays it is playing pivotal role as it is relatively non-toxic, efficient, and inexpensive. Three step essentially comprise the reactions which occur on the surface of photocatalyst: (i) absorption of the light, (ii) disconnection and transfer of photogenerated electrons, (iii) and redox reaction. The main argument about the dye degradation mechanism is that oxygen and superoxide radicals are produced from the reaction of photogenerated electrons and hydroxyl radicals which then degrade the methylene blue (MB) dye. The CuO–NH_2_ (nano)particles photocatalytic activity have been screened by the degradation of methylene blue. The prepared (nano)particles were transferred to the dye-containing solution, and the mixture was irradiated by the lamp, as mentioned earlier (visible light)^67^. The UV–Vis spectra (Fig. 6A) revealed that the dye degradation (decrease in maximum absorbance) of MB in the presence of synthesized (nano)particles (CuO–NH_2_) occurred after 80 min with 97.4% efficiency which is significant result among all CuO-based nanoparticles. Based on the outcome of studies, the time and the yield of degradation process by aminated CuO were found to be the best (Table 3)^98^.

**Table 3:** Recently reported photocatalytic activity of various plant extract mediated CuO nanoparticles.

Source	Dye	Time (min)	Efficiency (%)	References
Visible light	Rhodamine B (RB)	180	91	^44^
Visible light	Methylene blue (MB)	150	77	^100^
Hg lamp λ = 365	Methylene blue (MB)	120	79.11	^101^
Visible light	Crystal violet (CV)	300	97	^93^
Sunlight UV light	Methylene blue (MB)	120	96.9	^101^
125 W UV lamp	Methylene orange (MO)	180	94.4	^102^

In this work, before performing the photocatalysis process in presence of light, we screened CuO–NH_2_ photo degradation efficiency under dark condition (Fig. 6B). Under dark conditions, it caused only ~ 7% degradation after 80 min. On the other hand, in the presence of light, more than 97% degradation is recorded thus clarifying that the light is responsible for the degradation of methylene blue^99^.

### Photodegradation process for methylene blue (MB)

**Figure 7:**
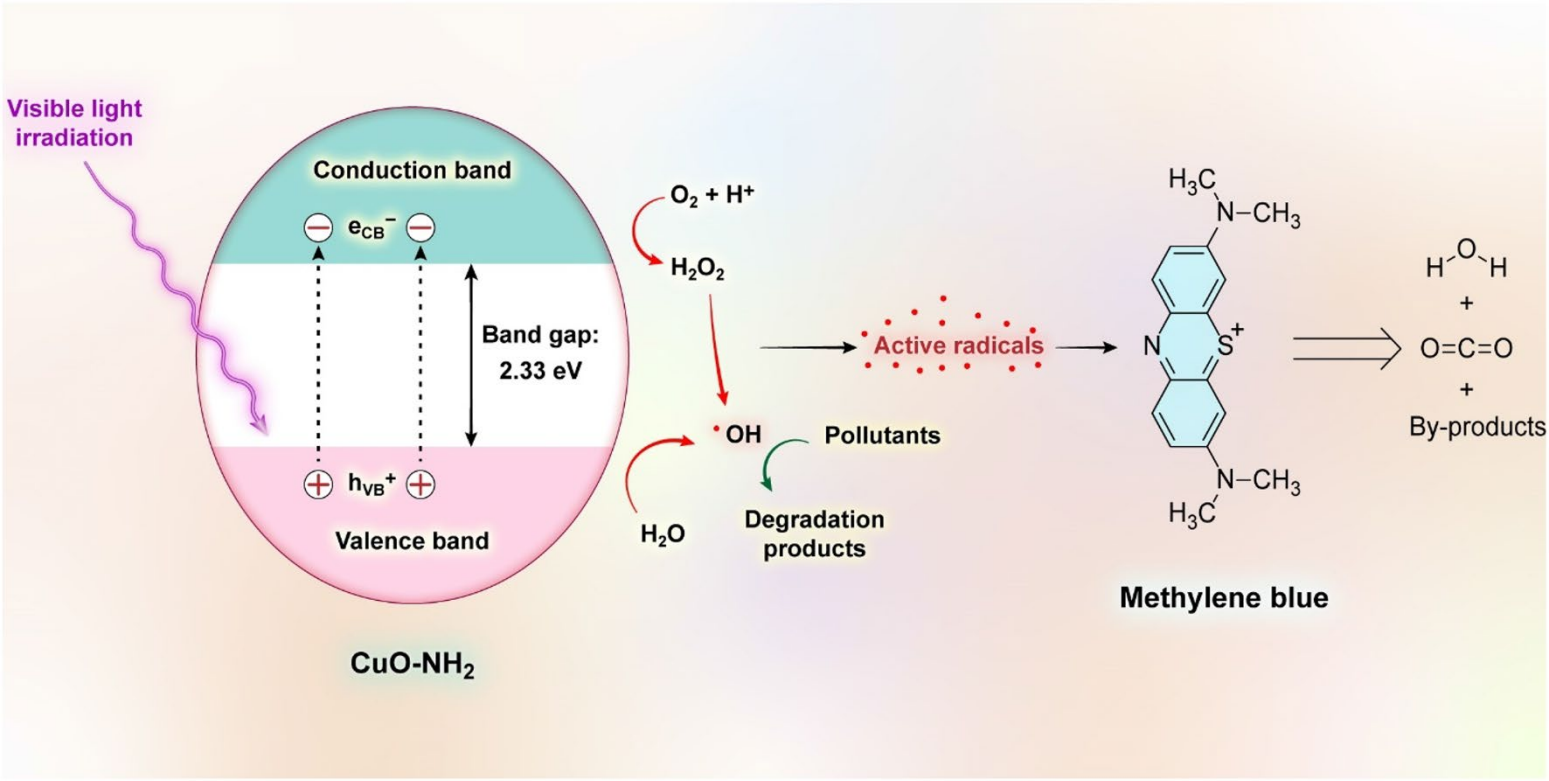
Schematic for the MB photodegradation mechanism.

The plausible photocatalytic mechanism for the photodegradation of MB dye by synthesized CuO–NH_2_ NPs is presented in Fig. 7. Irradiation of visible light to the CuO–NH_2_ NPs' catalytic surface causes the movement of photoexcited electrons from the VB to the CB and produces electron–hole pairs, as displayed in Eq. (1):

1 $${\text{CuO}} + {\text{h}}\nu \to {\text{CuO }} ( {{\text{h}}^{ + }_{{{\text{VB}}}} + {\text{ e}}^{ - }_{{{\text{CB}}}} } )$$

Furthermore, as light-excited electrons are transferred from the VB to the CB, an equal number of holes are created in the VB. The oxidation process for water molecules occurs by the valence band and causes the production of active OH free radicals, as displayed in Eq. (2):

2 $${\text{H}}_{{2}} {\text{O }} + {\text{h}}^{ + }_{{{\text{VB}}}} \to {\text{ H}}^{ + } + {\text{ OH}}^{ \cdot }$$

In the conduction band, due to the reduction process of the oxygen molecules, superoxide free radicals are produced, and finally, these produced radicals are converted into hydroxyl free radicals in several consecutive steps, as displayed in Eq. (3)^103^:

3 $${\text{O}}_{{2}} + {\text{e}}^{ - }_{{{\text{CB}}}} \to^{ \cdot } {\text{O}}_{{2}}^{ - } \to {\text{ HO}}_{{2}}^{ \cdot } \to {\text{ H}}_{{2}} {\text{O}}_{{2}} \to^{ - } {\text{OH }} +^{ \cdot } {\text{OH}}$$

Eventually, the photodegradation of desired dye occurs via the produced active ·OH free radicals, as displayed in Eq. (4):

4 $$^{ \cdot } {\text{OH }} + {\text{ MB }} \to {\text{ H}}_{{2}} {\text{O }} + {\text{ CO}}_{{2}}$$

The photodegradation of MB dye entails several steps up to generate the H_2_O and CO_2_ molecules eventually. The photodegradation of organic pollutants is accomplished by powerful oxidants such as ·OH radicals, which are shown in Fig. 7^104^.

### Cytotoxicity

**Figure 8:**
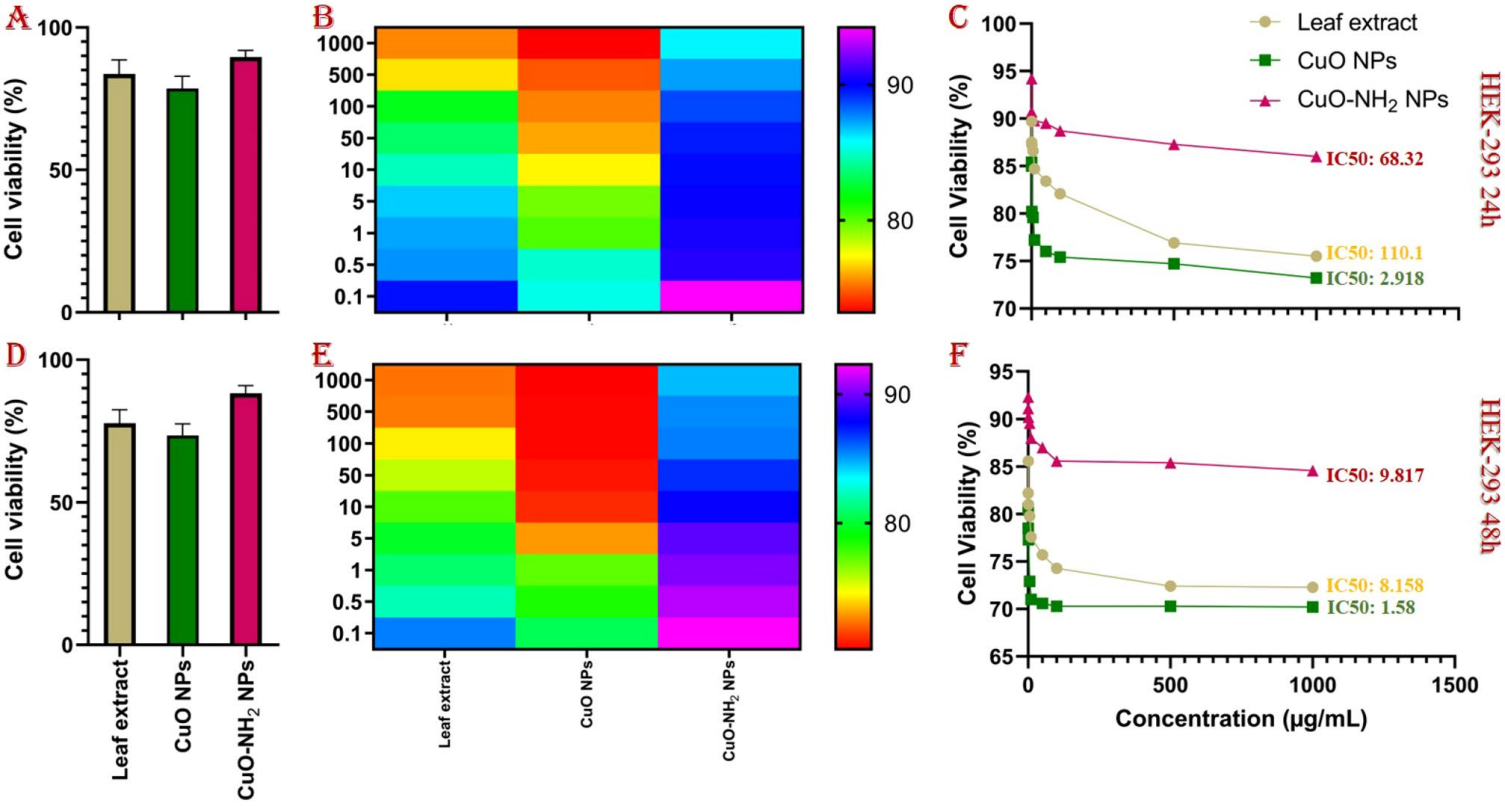
The average results of MTT assay after 24 h (**A**) and 48 h (**D**) of treatment on HEK-293 cell line. The heat map graphs of MTT assay with various concentration from 0.1 to 1000 µg/mL of different (nano)materials on HEK-293 after 24 h (**B**) and 48 h (**E**). The dose-dependent MTT assay results and IC-50 values on HEK-293 after 24 h (**C**) and 48 h (**F**) treatment.

It is obvious that the prepared (nano)material’s cellular safety must be considered in advance of any potential biomedical appliance. The cytotoxicity of copper oxide nanoparticles has been reported in some papers, but the lack of utilization of a green method could increase the cytotoxicity. Based on other studies, the cytotoxicity of *Rosmarinus officinalis* leaf’s extract was quite low and the cell viability was relatively high. On the contrary, the cytotoxicity of CuO (nano)particles is higher and their cell viability is lower. Based on the zeta potential results, the surface amination has led to considerable increases in surface potential (Figs. S3 and S4)^105^. It is evident that by making the surface potential more positive, the cytotoxicity of compounds will drastically decrease and The MTT assay results of aminated product clearly has affirmed this claim. The more positive surface, makes the prepared compound a good candidate for drug and gene delivery. So, the proposed method (surface amination) can be utilizable in various nanosystem for biomedical purposes. The cell viability of CuO–NH_2_ is conspicuously higher (~ 21%) while the cytotoxicity is considerably lower. The prepared (nano)material’s stability and safety has been confirmed by convergent trend results of 24- and 48-h treatment (Fig. 8A,D). Also, the heat map graphs are depicting the interrelationship among (nano)materials various concentration and relative cell viability (Fig. 8B,E). The dose-dependent response of prepared (nano)materials is proved by MTT assay with utilized the (nano)materials at varying concentrations namely 0.1, 0.5, 1, 5, 10, 50 µg/mL; Fig. 8C,F depicts the IC-50 values^73,89,106,107^.

## Conclusion

The present study focuses on the biosynthesis of aminated copper oxide (nano)particles from *Rosmarinus officinalis* leaf extract for the first time wherein an inclusive study about the potential biological and photocatalytic activity was undertaken. The prepared (nano)particles have been fully characterized and revealed a promising photocatalytic activity on degradation of methylene blue dye (~ 97% degradation under light in 80 min and just ~ 7% in dark condition and 80 min). These biosynthesized (nano)particles displayed potential antibacterial activity as well when screened against *Bacillus* as a gram-positive and *Pseudomonas* as a gram-negative bacterium (~ 50% increase in antibacterial activity for CuO–NH_2_ compared to CuO (nano)particles). The encouraging results are due to the synthesis technique deployed for the metal oxide nanoparticles and, specifically, the first aminated copper oxide (nano)particles with this promising potential. It is quotable that the amination of CuO led to considerable increase (Δζ = + 13.4 mV) in zeta potential of prepared (nano)particles. Additionally, the MTT assay investigation of the biosynthesized aminated copper oxide (nano)particles revealed that the proposed modification method leads to lower cytotoxicity (~ 21%). Owing to the considerable stability of prepared nanoparticles in greener media and based on remarkable results in vitro studies, the impressive ensued biological activity are additional attractive features of this study.

## Supplementary Information

Supplementary Figures.

## Data Availability

All data generated or analyzed during this study are included in this published article.
