# Supplementary material for: Bioengineering of CuO porous (nano)particles: role of surface amination in biological, antibacterial, and photocatalytic activity
Source: Sci Rep. 2022 Sep 12;12:15351. doi: 10.1038/s41598-022-19553-2 (PMC9467996; doi:10.1038/s41598-022-19553-2)
Supplement: Supplementary file 1 — Supplementary Figures. [file 41598_2022_19553_MOESM1_ESM.docx]

**Bioengineering of CuO porous (nano)particles: Role of surface amination in biological, antibacterial, and photocatalytic activity**

**Mojtaba Bagherzadeh^1^*, Moein Safarkhani^1^, Amir Mohammad Ghadiri^1^, Mahsa Kiani^1^, Yousef Fatahi^3,4,5^, Fahimeh Taghavimandi^1^, Hossein Daneshgar^1^, Nikzad Abbariki^1^, Pooyan Makvandi^6^, Rajender S. Varma^7^, Navid Rabiee^2,8^**

1. Department of Chemistry, Sharif University of Technology, Tehran, Iran
2. School of Engineering, Macquarie University, Sydney, New South Wales, 2109, Australia
3. Nanotechnology Research Centre, Faculty of Pharmacy, Tehran University of Medical Sciences, Tehran, Iran
4. Department of Pharmaceutical Nanotechnology, Faculty of Pharmacy, Tehran University of Medical Sciences, Tehran, Iran
5. Universal Scientific Education and Research Network (USERN), Tehran, Iran
6. Istituto Italiano di Tecnologia, Centre for Materials Interfaces, Pontedera 56025, Pisa, Italy
7. Regional Centre of Advanced Technologies and Materials, Czech Advanced Technology and Research Institute, Palacky University, Olomouc, Slechtitel, ů 11, 783 71, Olomouc, Czech Republic
8. Department of Materials Science and Engineering, Pohang University of Science and Technology (POSTECH), 77 Cheongam-ro, Nam-gu, Pohang, Gyeongbuk, 37673, South Korea

***Corresponding author**: Prof. M Bagherzadeh (bagherzadeh@sharif.edu)


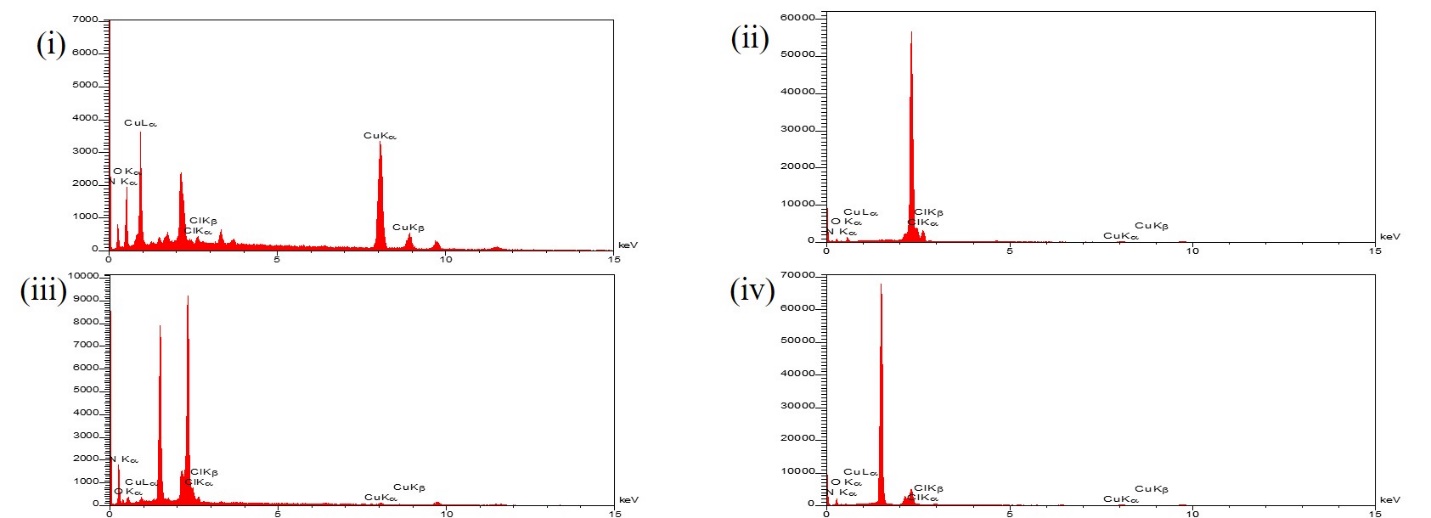


**Figure S1**. The EDS graph of prepared nanomaterials i) CuO, ii) CuO-Cl, iii) CuO-NH_2_, and iv) (CuO-NH_2_)’.


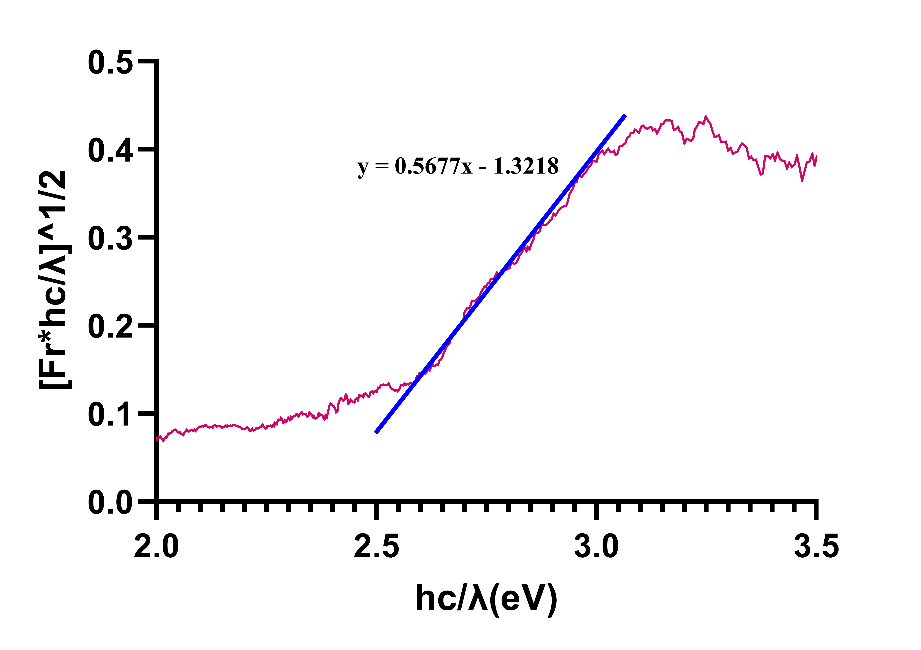


**Figure S2**. The Tauc plot of CuO-NH_2_.

**Table S1.** The IC-50 value of HEK-293 24h.

|  | Leaf extract | CuO NPs | CuO-NH_2_ NPs |
| --- | --- | --- | --- |
| Inhibitor vs. response |  |  |  |
| Bottom | 74.44 | 74.64 | 86.25 |
| Top | 87.61 | 85.39 | 91.37 |
| IC50 | 110.1 | 2.918 | 68.32 |
| logIC50 | 2.042 | 0.4651 | 1.835 |
| Span | 13.17 | 10.75 | 5.124 |
| 95% CI (profile likelihood) |  |  |  |
| Bottom | 68.87 to 77.67 | 72.56 to 76.49 | -infinity to ??? |
| Top | 86.14 to 89.16 | 82.44 to 90.14 | 89.61 to ??? |
| IC50 | 32.35 to 415.9 | 0.5403 to 15.30 | ??? to +infinity |
| logIC50 | 1.510 to 2.619 | -0.2674 to 1.185 | ??? to +infinity |
| Goodness of Fit |  |  |  |
| Degrees of Freedom | 6 | 6 | 6 |
| R squared | 0.9541 | 0.9254 | 0.7241 |
| Sum of Squares | 8.638 | 11.49 | 11.69 |
| Sy.x | 1.2 | 1.384 | 1.396 |
| Constraints |  |  |  |
| IC50 | IC50 > 0 | IC50 > 0 | IC50 > 0 |
| Number of points |  |  |  |
| # of X values | 9 | 9 | 9 |
| # Y values analyzed | 9 | 9 | 9 |

**Table S2.** The IC-50 value of HEK-293 48h.

|  | Leaf extract | CuO NPs | CuO-Cl NPs |
| --- | --- | --- | --- |
| Inhibitor vs. response |  |  |  |
| Bottom | 72.92 | 70.11 | 85.12 |
| Top | 83.72 | 81.32 | 91.58 |
| IC50 | 8.158 | 1.58 | 9.817 |
| logIC50 | 0.9116 | 0.1986 | 0.992 |
| Span | 10.8 | 11.21 | 6.46 |
| 95% CI (profile likelihood) |  |  |  |
| Bottom | 70.55 to 74.91 | 69.72 to 70.49 | 83.99 to 86.10 |
| Top | 81.42 to 86.72 | 80.49 to 82.22 | 90.50 to 92.80 |
| IC50 | 1.581 to 39.57 | 1.162 to 2.154 | 3.218 to 32.09 |
| logIC50 | 0.1989 to 1.597 | 0.06513 to 0.3332 | 0.5076 to 1.506 |
| Goodness of Fit |  |  |  |
| Degrees of Freedom | 6 | 6 | 6 |
| R squared | 0.9403 | 0.9956 | 0.9594 |
| Sum of Squares | 10.19 | 0.5936 | 2.445 |
| Sy.x | 1.303 | 0.3145 | 0.6384 |
| Constraints |  |  |  |
| IC50 | IC50 > 0 | IC50 > 0 | IC50 > 0 |
| Number of points |  |  |  |
| # of X values | 9 | 9 | 9 |
| # Y values analyzed | 9 | 9 | 9 |


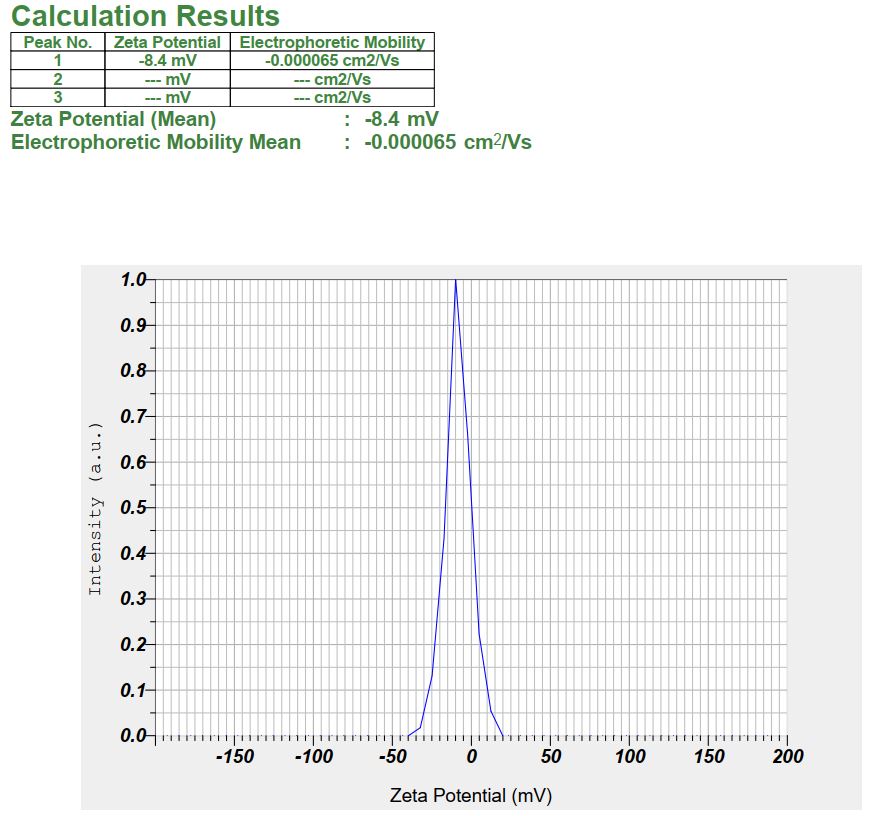


**Figure S3.** The zeta potential of CuO (nano)particles.


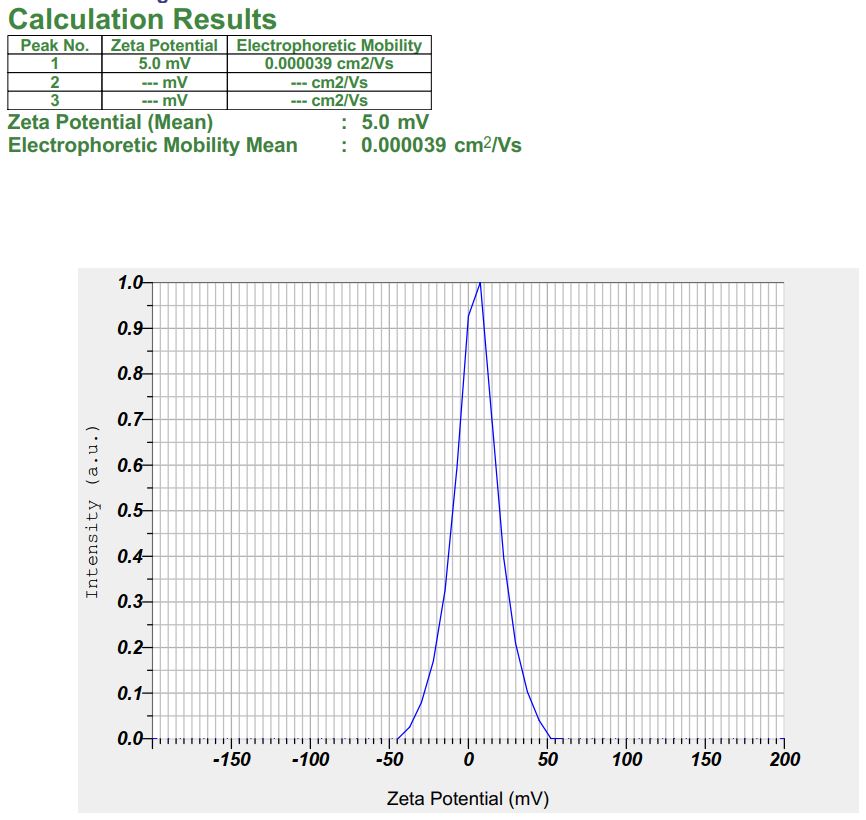


**Figure S4.** The zeta potential of CuO-NH_2_ (nano)particles.


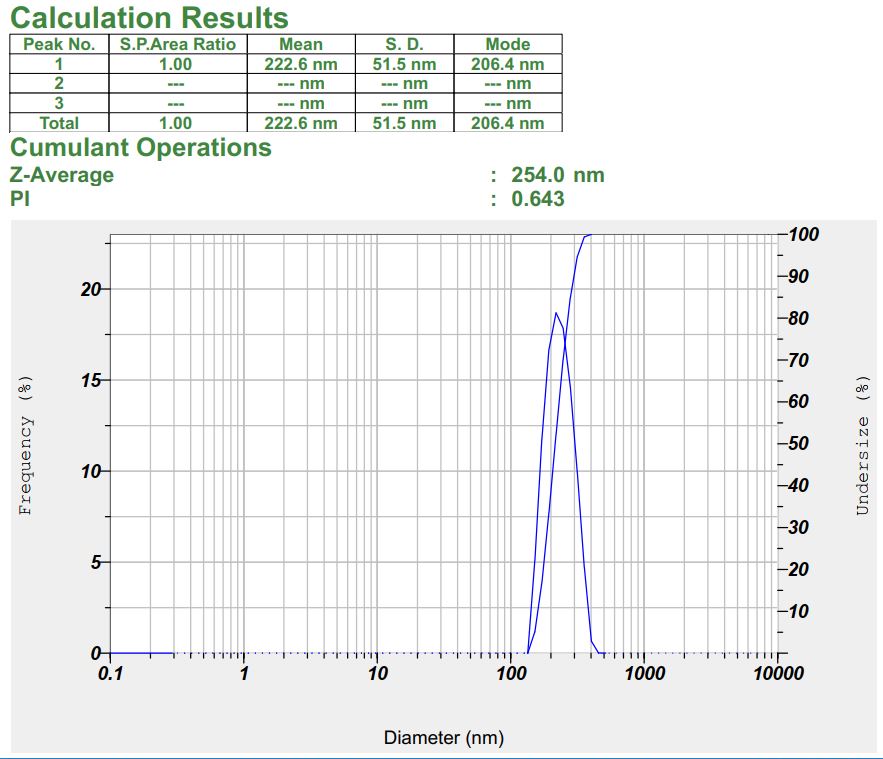


**Figure S5**. The DLS result of CuO (nano)particles.


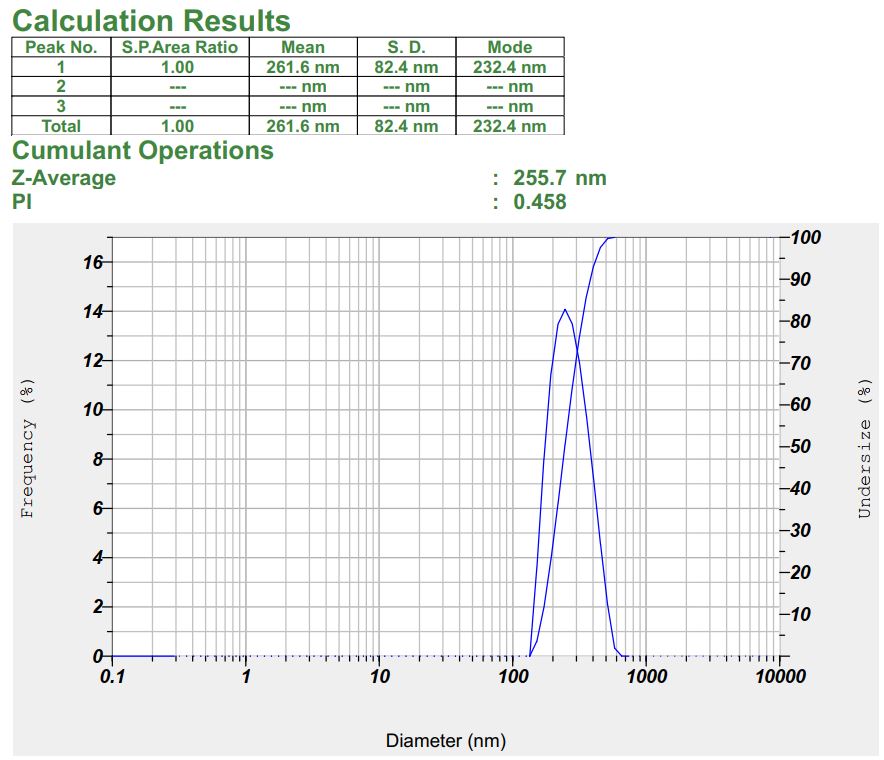


**Figure S6**. The DLS result of CuO-Cl (nano)particles.


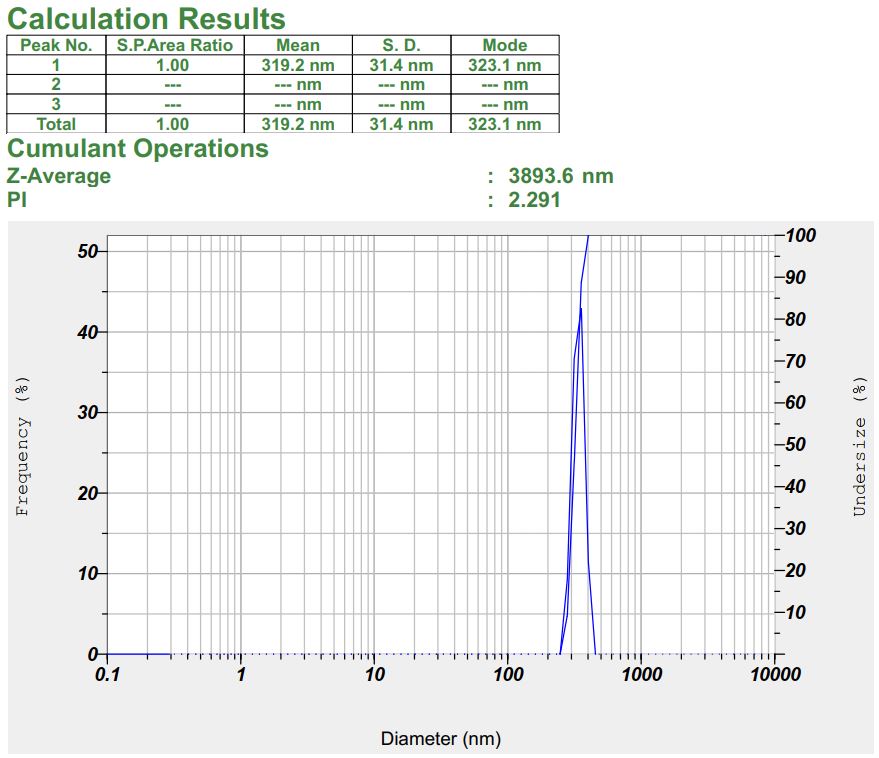


**Figure S7**. The DLS result of CuO-NH_2_ (nano)particles.
